# Addressing scabies among street children in Ethiopia: an ethnographic study of acceptable interventions by prospective recipients and deliverers

**DOI:** 10.3389/fpubh.2025.1529012

**Published:** 2025-04-09

**Authors:** Bewunetu Zewude, Desta Ayode, Gail Davey, Shahaduz Zaman, Getnet Tadele

**Affiliations:** ^1^Department of Sociology, College of Social Science and Humanities, Wolaita Sodo University, Sodo, Ethiopia; ^2^Department of Sociology, College of Social Sciences, Addis Ababa University, Addis Ababa, Ethiopia; ^3^Centre for Global Health Research, Brighton & Sussex Medical School, Brighton, United Kingdom; ^4^School of Public Health, Addis Ababa University, Addis Ababa, Ethiopia

**Keywords:** acceptability, interventions, scabies, street children, Ethiopia

## Abstract

**Introduction:**

Scabies is a neglected tropical disease that affects the physical, socioeconomic, and psychological wellbeing of patients. Street children, due to poor living conditions and social marginalization, are at increased risk of infestation and face significant barriers to access healthcare services. Various interventions to control scabies have been suggested and implemented, but few have been based on the needs and viewpoints of the street children themselves. Drawing on the theoretical framework of acceptability (TFA), this article explores the interventions that street children, parents, and other key informants perceived to be appropriate to control scabies among street children.

**Methods:**

An ethnographic approach was adopted to collect qualitative data from purposively selected street children, parents/caregivers, and key informants representing NGOs and the health and social sectors in Addis Ababa, Hawassa, and Adama. Rich data were gathered using FGDs, in-depth interviews, key informant interviews and drawing exercises, enhancing children's engagement in the study. Interviews were conducted in participants' preferred languages, recorded, transcribed verbatim, and translated into English for analysis. Data were coded by the two researchers who had collected them, and themes and sub-themes were identified.

**Results:**

Living in overcrowded conditions, lack of access to sanitation materials and health services were among the priority needs and lived experiences of the street children. Providing peer-led health education, educating and mobilizing existing healthcare providers, preventing child streetism, raising the awareness of the community children migrate from, and providing sanitation facilities were among the proposed interventions that were considered appropriate to control scabies among street children.

**Conclusion:**

Collaborative and participatory interventions that align with the lived experiences of street children and other stakeholders are likely to increase participation and enhance the feasibility and impact of scabies control and elimination efforts.

## Background

Scabies is a neglected tropical disease that affects more than 400 million people worldwide annually (1). Recent trends indicate a resurgence in scabies prevalence, suggesting a persistent gap in global prevention and treatment efforts (2). Low economic status, unhygienic behavioral practices, being a patient in a long-term care facility, frequent patient contact, lack of personal protective equipment, sharing personal materials, and having contact with others have been identified as risk factors (3–5). Populations most at risk of infestation include people living in overcrowded environments, refugees, displaced individuals, prisoners, and those with inadequate access to hygiene facilities. Children, older people, frequent travelers, resource poor endemic populations, and the immunocompromised are also highly vulnerable (6–10).

Various intervention strategies aim to control scabies infestation and reduce its impact on the quality of life of patients. These include mass treatment of patients with permethrin cream or ivermectin (11, 12), massive public mobilization and public health emergency interventions (13), improvements in primary care and public health management (14), providing medical treatment, breaking the chain of transmission, source elimination and disinfection of fomites (12). The importance of including non-medical personnel such as teachers, cadres, and parents alongside local health workers, ensuring cooperation between patients, their families, health workers and other non-medical personnel has also been emphasized. Establishing a sustainable drug supply and fostering partnerships and meaningful collaboration among stakeholders was thought to be critical for progress toward control of scabies (11). According to Thompson et al. (9), the early identification of patients with scabies and treatment of their contacts reduces community transmission.

Scabies is a common public health concern in Ethiopia, affecting more than one million people with a prevalence of more than 10% in some *woredas* (districts) (2, 13). Despite being a recognized public health issue in Ethiopia, marginalized groups like street children remain understudied in scabies-related research, creating a critical gap in intervention strategies. The target populations of scabies-related studies conducted in Ethiopia and elsewhere in the world predominantly constitute children (15–17) or adults (18–20) who are part of mainstream society. The marginalized segments of society, including street children who are highly vulnerable to the condition (10, 21, 22) have been neglected. Most studies have been quantitative cross-sectional surveys, suggesting a need to use social science vantage points supported by ethnographic methods with emic perspectives.

This study was guided by the Theoretical Framework of Acceptability (TFA). Sekhon et al. (23) defines acceptability as a multi-faceted construct that reflects the extent to which people delivering or receiving a healthcare intervention consider it to be appropriate, based on anticipated or experienced cognitive and emotional responses to the intervention. The framework provides a structured, evidence-based guide for assessing the acceptability of healthcare interventions from the perspectives of people receiving or delivering interventions (24). It originally consisted of seven component constructs: affective attitude, burden, perceived effectiveness, ethicality, intervention coherence, opportunity costs, and self-efficacy (23). Casale et al. (25) later added other factors – (1) the extent to which young people consider the intervention to be relevant to their needs and lived experiences, (2) perceived positive and negative consequences of the intervention, and (3) perceived social acceptability. Paynter et al. (24) also added ‘perceived safety and risk' in relation to acceptability of surgical interventions.

According to Paynter et al. (24), TFA has rapidly been adopted in healthcare research and used to inform data collection and analysis. For instance, Pavlova et al. (26) revealed that women in England accepted a postnatal walking intervention because meeting other mothers and being physically active aligned with their value system and attending the group required little effort. Abamecha et al. (27) explored the acceptability and feasibility of the school-engaged social and behavior change communication approach on malaria prevention in Ethiopia and found that self-efficacy, community support, school climate, perceived threat, and knowledge affected acceptability of the approach. Casale et al. (25) found that the acceptability of healthcare and social interventions among young people in Africa was determined by understanding the intervention, ease of use, intervention costs and barriers to access, perceived positive and negative effects of the intervention, relevance to the young people's needs, and prior engagement with the intervention.

This article is based on the assumption that understanding the interventions that are acceptable for both prospective deliverers and recipients and designing interventions accordingly enhances participation and effectiveness of interventions. Understanding which interventions are acceptable to both intervention deliverers and recipients helps to highlight which aspects of the intervention need modification to increase acceptability and participation (23). Perski and Short (28) also contend that acceptability has the ability to predict and explain key outcomes of interest, including user engagement and intervention effectiveness. Drawing from the concepts and assumptions of TFA, this article explores which prospective interventions to address the burden of scabies among street children in Ethiopia might be acceptable to street children, parents or caregivers, and key informants (both prospective service recipients and service deliverers).

## Materials and methods

### Study design

The study used an ethnographic design to explore the perspectives of street children and key stakeholders. Ethnographic methods offer deep insights into the lived experiences of marginalized populations, making them particularly suitable for understanding scabies interventions.

### Study area

The study was undertaken in three Ethiopian cities, Addis Ababa, Hawassa, and Adama. Previous studies (29–32) revealed the high concentration of street children in these urban areas, and the multifaceted health and other problems they face. Because many of these children migrate from the small rural villages surrounding these urban areas (33), the study benefited from their diverse perspectives and lived experiences, reflecting variation in their cultural and socioeconomic backgrounds.

### Study population and sampling

The study targeted street children in three cities mentioned above. Purposive sampling method was used to select study participants. Both children of the street (unsupervised children relying on the street for shelter and livelihood) and children on the street (semi-supervised children who return home at night) (34) were included. In addition, parents or caregivers living or working on the street, health professionals and representatives of NGOs working with street children-related programs/projects, officials from the health bureau and the bureau of women and social affairs (BoWSA) also participated. Snowball sampling was also used to identify under-represented groups (female street children and male parents/care givers), often by engaging the informal “bosses” on the street.

The major inclusion criteria were age 7–15 years, living/working on the street, at least 2 months since joining street life, and willingness to participate in the study. Children from 7–15 were targeted because evidence indicate that this age group is most affected by scabies and its consequences (15, 35). We entertained flexibility in our inclusion/exclusion criteria to allow maximum variation in the characteristics of the study participants, including age, sex, level of education, number of years living/working on the street, level of attachment to the street, and experience of infestation with scabies. Consequently, 36 street children (12 from each city) participated in the in-depth interviews (IDIs) and another 30 in focus group discussions (FGDs). There was 1 male FGD and 1 female FGD in each city, with each FGD containing 5 participants. Twenty-seven participants were included in the KIIs (5 parents or care givers, 1 health professional, 1 health bureau representative, 1 from BoWSA, and 1 NGO representative working on street children, from each of the three cities). The number of study participants was determined by the principle of theoretical saturation; interviews were conducted until the point where the researchers realized that no additional themes or insights emerged from the data collection, and all conceptual categories had been explored, identified, and completed (36).

### Method of data collection

A triangulated approach, incorporating multiple methods, data sources, and researchers, enhanced the depth and validity of the findings (37, 38). We used in-depth interviews (IDI), key informant interviews (KII), focus group discussions (FGD), and observations. In a bid to enhance children's engagement in the study, we used a participatory method, specifically drawing exercises. The two multidisciplinary researchers who collected the data had relevant professional backgrounds and previous experience in the same settings and study population.

All the data collection tools were first prepared in English, translated to the local languages of the study participants and then back translated. Among others, the major questions we asked included: “What intervention/s do you think will help address the problem of scabies among street children?,” “To what extent do you think the proposed intervention will be effective?,” “Do you have previous experience of implementing a similar/related intervention with street children or other disadvantaged groups?,” and “What enabling conditions exist to successfully implement the proposed intervention?” In all study sites, study participants were interviewed in their own [preferred] mother-tongue language. Undertaking the interviews in the natural setting where the children lived, worked, and interacted among themselves gave us the chance to observe and understand the context of their vulnerability to scabies.

The data collection took place from March to May, 2024. The average length of interview varied; for the street children, individual interviews lasted from 23 min (minimum) to 43 min (maximum). While this variation primarily depended on the children's age and ability to express their views, we aimed to keep the interviews as brief as possible to maintain their attention. We arranged private, convenient locations, mainly the compounds of churches and NGOs providing rehabilitation services. Key informants were contacted in their offices. With the consent/assent of the study participants, all the interviews were audio recorded and these were supplemented with field notes.

### Method of data analysis

We used thematic analysis with a hybrid approach of inductive and deductive coding and theme development (39). All audio records were first transcribed verbatim and then translated to English by the two authors who collected the data. After deep reading, initial codes were manually developed independently and reviewed collaboratively. The other authors familiarized themselves with the data and context through debriefing sessions conducted at intervals through the lifetime of the project. We created a coding book on google docs and made it accessible to all authors to be able to comment, add, marge, or remove the codes that emerged both from the fieldnotes and later from the transcripts. Coding was followed by generating themes and sub-themes, reviewing potential themes iteratively, and defining and naming themes. The results were presented in the form of narratives where key findings were supported by selected quotes.

### Ethical considerations

Ethical approval was obtained from the Institutional Review Board of the Ethiopian Society of Sociologists, Social Workers, and Anthropologists (ESSSWA) in Ethiopia (Ref: ESSSWA/L/AA/0536/2024). Participants were informed that their participation was voluntary and that they were free to withdraw from the study at any time without being afraid of any possible consequences. Informed consent and assent processes were tailored to participants' unique circumstances, ensuring ethical participation. This way, children who were not attended by parents provided assent for participation, while parents were asked to provide their consent for those who had contact with their families. All the data were stored securely. In the report, we have deidentified all participants and, where mentioning personal identifying characteristics such as age and sex was necessary, we used pseudonyms. We provided moderate incentives, such as inviting them lunch, to compensate for the time spent during data collection. Referral pathways were established for children with severe scabies cases, and those seeking rehabilitation services were assisted in accessing appropriate programs.

## Results

Scabies was identified as a prevalent health issue among street children across all study sites, significantly impacting their physical, social, and psychological wellbeing. Due to severe resource constraints, street children faced poor living conditions that heightened their vulnerability to scabies and other infectious diseases. Limited access to healthcare and social services further hindered their ability to respond to outbreaks. Their lack of health literacy, shaped by low educational attainment and the absence of targeted health education campaigns, also contributed to the persistence of scabies among them. Based on these challenges, several interventions were proposed by participants.

### Educating and mobilizing existing healthcare providers

Highlighting the presence of an existing system as a facilitating factor, key informants recommended strengthening the healthcare system by providing relevant training to health extension workers. This training would empower them to deliver targeted health education and promote health-seeking behavior among street children. Additionally, mobilizing the healthcare workforce through advocacy and sensitization programs was suggested.

There is no special system or service designed for street children. Health extension program is expanding everywhere but has not considered the needs of street children. It could be more effective to use health extension workers for campaigns to deliver health education for street children in various corners. (key informant, NGO)

Among the four categories in our health extension program is providing healthcare services to the homeless. Health extension workers could be trained and oriented to add communicable diseases such as scabies to their outreach health education activity. In addition to creating awareness, they should make patients affected by the condition to report to healthcare facilities and receive treatments. (key informant, health bureau, Adama)

There is an already established system such as a rapid response team within the health bureau that can respond to scabies if needed. You don't therefore need to create something new; you rather need to focus on the existing structure and strengthen it. If they (government) are interested, the issue is not beyond the capacity of the health system. Budget is not an issue too; health centers are free to decide on their internal budget. The same way they allocate certain amount of budget for HIV or other conditions, they can also do the same for scabies. I am sure that they can handle scabies without the need for support from external sources. (key informant, health bureau, Addis Ababa).

### Providing health education through peers and other communication methods

Given that many of the children had little formal education and lacked health literacy, the need to raise their awareness about the prevention and treatment of scabies through peer-led health education campaigns was suggested.

Recruiting peer educators from among street children is a more effective strategy. Training them on scabies and communication techniques enables them to better engage with other street children. Peer educators make it easier to connect with this population. (Key informant, NGO)

The need for health education has also been emphasized because misconceptions about the epidemiology of scabies, superstitious thinking, and poor health seeking behavior predominate among the street children.

I have never visited a health facility and I don't want to visit for scabies. It is only if I am infected by another disease that I visit a healthcare facility, not because of scabies. It will disappear by its own time. (street child, 14, M)

I don't think you can prevent scabies unless God protect you. (street child, 15, M)

When they hate me because of my scabies, they will be infested soon. If someone sees your scabies and hated you because of your condition, he will soon contract the scabies. (street child, 13, M)

Drawing from the experience of implementing interventions against HIV/AIDS in other vulnerable populations, some key informants argued that implementing peer education programs would help control scabies and other communicable diseases among street children. The fact that street children have many shared attributes and face common problems was thought to add to the effectiveness of peer education sessions.

We have the experience of working on HIV prevention among commercial sex workers. We have been achieving this through peer education sessions. Because these peers mostly belong to similar age groups and experience the same problems, creating sessions to discuss their common problems will help generate effective and acceptable solutions. Similarly, if you first train some active members that could facilitate the sessions and enable the street children to discuss scabies related issues in peer sessions, I think this will help manage the condition. The street children have diverse education backgrounds that range from those who have never attended school to university graduates. This gives you the chance to select and train the better educated ones that will later train others during the peer discussion sessions. This way they can discuss about scabies in their own settings such as while chewing khat. Making the government to take ownership of the intervention will also help to make it sustainable. (Key informant, health bureau, Adama)

Study participants also expressed their readiness to learn about scabies believing this would assist them to protect themselves. Many street children commented on the absence of such interventions before and expressed their willingness to participate in health education programs.

….by the way, in the name of all street dwellers, I am very thankful for your understanding and raising this concern. We are very happy if we get the opportunity to learn about this disease. We are highly vulnerable to scabies and severely affected when it spreads. Scabies is a common health issue among street dwellers, and it is likely to infect many people once it emerges. This is because we do many activities collectively—eating, sleeping, and other daily tasks are often done in groups. (Parent, 36, M)

I would suggest that the main thing is children should be educated about scabies so that they would take more care about their personal hygiene. Providing sufficient information for street children is important. (street child, 15, M)

The need for using infotainment as a strategy of providing health education was suggested as a way of enhancing children's engagement.

Sensitization campaigns should be accompanied by entertainment programs as music and drama can attract the attention of the children to receive messages relevant for addressing scabies. (key informant, NGO)

In relation to peer-led interventions, using street subculture was considered instrumental to enhancing the participation of children through their informal leaders.

It is also a good idea to engage street children in the planning and implementation process. Working with the informal bosses can help facilitate their participation. (key informant, BoWSA)

A key informant from the health bureau argued that providing the children with health education or other services should be accompanied by further rehabilitation and reintegration interventions. He argued that educating the children and leaving them to continue in their usual living situations would not bring change.

Providing the street children health education alone may not be effective so long as they continue to live in the same situation that primarily puts them at risk of scabies. The government should collaborate in providing shelter where they could stay until they complete the treatment. They also need clothes, food, and water. If possible, it is also good if access to education and vocational training is created for them as it builds their future.

### The use of street children-friendly services to promote health seeking behavior

The children's living situations posed significant challenges in terms of adherence to interventions to prevent and treat scabies. The need to develop prevention and treatment strategies that fit their living conditions was suggested.

…when I went to the health center for scabies treatment, the health officer advised me to burn my clothes as much as possible or boil it with a very hot water. However, it was hard for me to adhere to his advice because I have no other clothes to change. Secondly, I don't have a place to boil the clothes as I am living on the street. Because of that, the treatment was not successful and I totally gave up applying the medicine. (street child, 15, M)

Adherence to the application of the ointment might be the biggest challenge when it comes to the street children due to their lifestyle. In fact, the gold standard treatment is the application of ointments on the skin. However, this line of treatment requires proper follow up and administration of the medicine and care. I would therefore suggest them to take tablet medicine which could be provided to children in two rounds and easy to administer and improves adherence. It is vital to consider this as an alternative treatment in mass administration. (key informant, Dermatologist)

A key informant from an NGO providing drop-in day care services for street children reported the experience of withdrawing from care and then coming back to the center with a relapse.

We observe adherence problems, especially among younger children who do not mostly treat scabies as a serious condition. While receiving treatment, they disappear for a while and come back with a relapse, seeking new treatment from the center.

Other study participants argued that the spatial mobility of the street children affects efforts to provide health services in accessible locations. Center-based treatment was thought to resolve this challenge.

I would suggest a center-based interventions. The first one is to establish a feeding and rehabilitation center and use these as a means to provide health education, for diagnosis and treatment of scabies, as well as rehabilitating them for reunification and reintegration. The fact that the children are mobile and live in various locations makes outreach interventions difficult. Therefore, center-based treatment could be the best option if that is implemented with voluntary admission and participation of the children. Secondly, it is possible to collaborate with NGOs providing center-based street children-related services if building a shelter is difficult. (Key informant, Dermatologist).

### Creating access to healthcare services for the children infested with scabies

Many street children and parents or caregivers assume that they are vulnerable to scabies because of their living conditions. They believe that a child infested with scabies should seek healthcare treatment as soon as possible since crowded living conditions facilitate transmission to other children.

…the best way to prevent scabies is to advise an infested friend to seek healthcare treatment. This is because, if he is treated and gets well, I will not be infested. (street child, 15, F)

… I advise anyone infested with scabies to seek treatments at the earliest possible opportunity. I will tell him that it is inevitable that will be transmitted to all of us unless he takes immediate measure. If he doesn't have money, we also support him by collecting it from our friends. (Parent, 36, M)

…I used to underestimate it. One of my friends who was severely affected by scabies advised me to take it seriously. My friends contributed money and bought me medicine although it aggravated my condition. When I told them the medicine was aggravating the condition, they advised me to go to sister's clinic. (street child, 13, M)

Street children did not have adequate access to healthcare services, especially from public health facilities. Creating access to health services was thought to address the burden of scabies on the street children.

Although there is Community-Based Health Insurance (CBHI) that is meant to create access to health care for the poor, street children do not have access to membership of this scheme. With the increasing number of street children at national level, a mechanism has to be in place for street children to benefit from CBHI. Therefore, without such insurance in place for impoverished member of the society, there is no way that street children will get access to free health care. Consequently, it is important that they should be provided free medical services. (Key informant, health bureau)

Organizations should provide healthcare service for the children infested with scabies. If they give them the medicine, I am sure they do not hesitate to receive it. I think everyone prefers care and relief to suffering from the condition. (street child, 15, M).

### Providing access to water, sanitation and hygiene facilities

The lack of facilities to maintain personal hygiene has been considered among the main reasons for street children's vulnerability to scabies. Participants therefore perceived that creating access to hygiene facilities will help to prevent scabies.

One way organizations can support street children could be to help them maintain their hygiene such as by providing them with the sanitation materials and clothes. Adjusting a place where the street children could take a bath and wash their clothes could be an alternative. Making the shower price cheaper may attract street children to frequently take shower and to be clean and healthy. (street child, 15, M)

The best intervention I could suggest is providing them sanitation materials. But, under conditions where the children do not get access to water to take a bath, this may not change the situation. Therefore, it is necessary to build such hygiene facilities in each sub-city in which the children can take a bath free of charge. Of course, this requires the combined efforts of the relevant stakeholders, such as the land administration as building the facilities requires land. (Key informant, BoWSA).

### Prevention of child streetism

Preventing children joining the street was suggested as an acceptable intervention to control scabies. Some participants believed that other interventions addressing the problem of child streetism might instead promote the problem as the availability of [free] services may attract other children to the street and those that are already on the street may not think to exit:

Strengthening the socioeconomic situations of families should be prioritized to prevent the children from joining the street. Sometimes, you will see that many of the interventions targeting street children such as reunification promote child streetism in the sense that the seed money given to the reunified ones motivates other parents to send their children to the street. Nevertheless, I don't believe that they should be denied access to these services because of such repercussions. Of course, I understand that this requires the coordination of various stakeholders. If it is well thought out, I think prevention is a viable strategy. (key informant, health bureau)

Trying to improve the living condition of the street children helps to prevent scabies. If possible, these children should sleep at home, resume school, and wear clean clothes. National level consultative workshops should be organized involving all the concerned stakeholders, including the regional administrators. This should be followed by an intervention to address the problem of child streetism mainly by working with the communities around the areas where most children come from. It is necessary to change the mindset of the community first. (Key informant, BoWSA).

### Rehabilitation, reunification and reintegration

Many street children believe that entering rehabilitation centers reduces their vulnerability to scabies and other health problems. Rehabilitation and reunification services could help address the problem of scabies among street children as these were believed to improve the conditions that put them at risk of scabies infestation. Street children also expressed their willingness to enter into these rehabilitation centers but also the discrepancy between demand for rehabilitation and access to these services.

Joining rehabilitation services will keep us from many risky behaviors, including addictions. If you enter in an organization that provides rehabilitation services, you will not be allowed to use addictive substances. What they (organizations) want is to see our lives changed and exit from the street life. They insist that we should stop using substances. (street child, 15, F)

There are so many children that want to join rehabilitation centers and hence organizations could easily ask and take them to the centers. (street child, 15, M)

It is better to stay and live with the family. In the family you will have a place to sleep, and you may not be exposed to cold, hunger, and health problems. (street child, 13, M)

Reintegration of street children and adolescents into the community was also believed to be an appropriate intervention to control scabies. Providing them access to various job and life skills training was thought to achieve successful reintegration of the children into the labor market and the community at large.

Street children should be supported to improve their livelihood and exit from the street. Instead of incarcerating them, the law enforcement bodies should support the children toward this goal. If the street children get the opportunity of learning life skills, they can get back to their normal life. But incarceration will not bring lasting solution. Had the government attempted to provide the street children with jobs and the children in turn hesitated to work, it could be fair to blame the street children and incarcerating them could be fair. The street children that can be able to reunite with their families should also be supported to be reunified. (parent, 27, F)

I don't think it is possible to completely stop the children from migrating to the street and not all children that have been reunified will be sustainably reintegrated into their family. It is therefore important to support and strengthen local NGOs working on street children. This will assist them to design and implement effective programs that would help the children learn various skills and ultimately be reintegrated into the labor market. Children that want to resume school could also be supported if they want to. (key informant, health bureau).

### Targeting source communities to enhance children's health literacy

Key informants argued that intervening in the source communities to raise health literacy of community members would help the children to prevent and respond to scabies. Therefore, educating the children and enhancing their health literacy while they are in their source communities was thought to reduce their vulnerability after they joined street life.

Campaigns targeting the source communities are important because many children come with scabies from the source communities. Hence targeting schools and the general population at the source community need to be considered if an intervention is to be effective. Using local radio and in a local language is also another area that should be given attention. (key informant, NGO)

Since children emerge from the community, it is essential to raise awareness while they are still part of it. Interventions in schools are also crucial, with teachers serving as key facilitators to promote hygiene and sanitation. Educating children in school ensures they retain awareness of sanitation, even if they later drop out and move to the streets. We adopted a similar approach during the outbreak. It is important to foster awareness and a sense of ownership within both the community and schools. By addressing the issue at its source, we can effectively reduce the burden on the streets. (key informant, health bureau, Hawassa).

### The need for inter-sectoral collaboration

Addressing the problem of scabies among street children through a single entity was considered ineffective and unsustainable. Coordinated efforts across a range of stakeholders, including the government, NGOs, and the private sector were suggested.

I believe that effective intervention could be possible by involving all relevant stakeholders, especially the health extension workers operating at grass root level and other health professionals supervising them. (key informant, health bureau)

It requires the contribution of many actors. The combined efforts of various stakeholders can make significant difference in addressing the problem of scabies. Sustainable change will be achieved through health extension program, and collaborative efforts of like-minded organizations. (Key informant, NGO)

Overall, the findings indicate that scabies remains a significant health issue for street children, exacerbated by poor living conditions, limited sanitation, and inadequate healthcare access. Proposed interventions include peer-led health education, improved sanitation facilities, and accessible healthcare services. Long-term solutions such as rehabilitation and reintegration, combined with multi-sectoral collaboration, are essential for sustainable change.

## Discussion

This study explored feasible interventions to control scabies among street children in Ethiopia, based on ethnographic data from three cities. The Theoretical Framework of Acceptability (TFA) has been used to organize and present the data. This discussion section critically examines the proposed interventions in the context of children's living conditions, barriers to care, and the involvement of key stakeholders. We evaluate how the proposed solutions align with the constructs of the TFA, such as ethicality, effectiveness, and feasibility from the perspectives of both service recipients and deliverers.

With slight variations across the regions and across study participants' characteristics, we found scabies was prevalent among the street children at all the study sites. Our findings align with those of Arnaud et al. (40) and Dixit and Shukla (41), who reported that street children's vulnerability to scabies is rooted in poor living conditions, overcrowded sleeping arrangements, and lack of hygiene facilities. However, while previous studies focused primarily on infrastructure deficits, our research highlights the role of children's limited health literacy and social exclusion as compounding factors. This suggests that addressing infrastructure alone may not be sufficient; targeted interventions to improve health education and access to healthcare services are essential.

Street children's vulnerability to scabies is related to their poor living and working conditions, as also demonstrated by other studies. Chowdhury et al. (42) found that poor living conditions such as unhealthy sleeping areas, irregular baths, little changing of clothes, and overcrowded living conditions contributed to street children's vulnerability to scabies infestation. Mukherjee et al. (43) also found high prevalence of scabies among street children with significant inter-group differences found by frequency of bathing, frequency of changing clothes and drug abuse. Arnaud et al. (40) revealed significant variations in scabies prevalence between the street homeless and the sheltered homeless populations, with higher prevalence among the former. The difference was linked to prevention and treatment measures, including access to on-site showers and laundries, and also better access to care and treatment.

The street children's suggestions reflect a strong sense of ethicality and relevance, as they understand that their vulnerability to scabies stems from their living conditions. Their positive affective attitudes toward sanitation facilities, combined with high self-efficacy, suggest that children believe they could effectively prevent scabies if provided with the necessary resources. This aligns with the TFA, which emphasizes the importance of aligning interventions with the values and capabilities of the target population to ensure engagement. From the point of view of the key informants from the health bureau, BoWSA, and NGOs (prospective deliverers), interventions that were perceived to be effective include enhancing the health literacy of the source community, creating free access to healthcare services, strengthening the health system, and preventing child streetism. Most importantly, the participation of various stakeholders was believed to enhance the effectiveness and feasibility of these and the other proposed interventions, as earlier argued by Abate et al. (44).

Peer-led health education interventions were proposed both based on perceived effectiveness and previous experience with successful HIV prevention campaigns. Peer-led interventions were thought to promote preventive and responsive behaviors of street children against scabies infestation, mainly by raising their health literacy. Consistent with our research, Arnaud et al. (40) suggest targeting prevention and awareness raising activities (including informing of the need to have one's own personal sleeping bag), and generalized distribution of hygiene kits containing suitable products and packaging to facilitate access to municipal baths/showers among street children in France. In a study undertaken in India, Mukherjee et al. (43) also suggested interventions that include improvement in personal hygiene and community awareness along with regular health checks, preventive measures in terms of health education and counseling to address the issues of drug abuse and sexual abuse and creating institutional linkages for the affected children. Therefore, clean and healthy lifestyle education should be repeatedly provided within short interval durations to remind the vulnerable population regarding the urgency of preventing scabies.

Both service recipients and deliverers identified rehabilitation, reunification, and reintegration as critical strategies to control scabies, emphasizing that stable housing improves access to healthcare and promotes treatment adherence. This finding is supported by Zewude et al. (45), who argued that reunifying children with their families helps address structural health challenges. However, our research suggests that without sustained support—such as vocational training and mental health services—reintegration efforts may fail, leaving children vulnerable to relapse into street life and reinfection. Brandenburg et al. (46) also suggested that quickly reuniting children with their primary caregiver is better for the mental health of the family, with the acute effects of community panic and upheaval reduced and the effects of post-traumatic stress minimized.

Vocational training plays a crucial role in successful reunification and scabies prevention by equipping children with skills to exit street life, thereby reducing their exposure to unhygienic environments. As Milton (47) argues, income-generating opportunities empower street children to sustain healthier lifestyles, minimizing their risk of reinfection. In addition to the interventions discussed above, many others have been suggested and some proved effective to address the problem of scabies and other communicable diseases among street children. Türkmen et al. (48) recommends establishment of day care centers where street children can obtain food and healthcare, empowering the family to reduce children's involvement in street life, and training law enforcement personnel to help in the fight against substance abuse.

In general, interventions aimed at addressing the problem of scabies among street children should be comprehensive and focus on changes in behavior and practice, both prevention and treatment services and management of risk factors (57). These services may include provision of free health care services in public health facilities (58), raising the awareness of street children about the causes, prevention mechanisms, and consequences of scabies and improving their sanitary behavior (49, 50), providing in-service trainings for the health work force (50, 51), community-wide interventions such as improving maternal education and caregivers' knowledge about scabies, ensuring safe water supply, providing adequate housing (16), mass drug administration and follow-up (52, 53), and sustainably reunifying and reintegrating them by providing effective rehabilitation services that include life skill training, psychosocial support, and initial capital (54, 55) as well as prevention of child streetism (56).

## Conclusion

Our findings highlight the need for targeted awareness campaigns tailored to the unique needs of street children, along with both institutional and outreach healthcare services. Policymakers should prioritize in-service training for healthcare providers to foster non-discriminatory practices and ensure street-friendly services. Additionally, multi-sectoral collaboration between health, social services, and NGOs will be essential for sustainable scabies control. Future research should explore the feasibility of integrating scabies treatment into existing healthcare programs and assess the long-term impact of rehabilitation interventions on children's health outcomes.

## Data Availability

The original contributions presented in the study are included in the article/supplementary material, further inquiries can be directed to the corresponding author.
